# Organic nutrient combinations for enhancing nutrient uptake and fruit quality in Sardar guava (*Psidium guajava* L.) cv. L-49

**DOI:** 10.3389/fpls.2026.1830499

**Published:** 2026-05-13

**Authors:** Reetika Sharma, Rakesh Kumar, Parshant Bakshi, Maanik

**Affiliations:** Division of Fruit Science, Sher-e-Kashmir University of Agricultural Sciences and Technology of Jammu (SKUAST-Jammu), Jammu, India

**Keywords:** guava (*Psidium guajava* L.), organic nutrient management, vermicompost, biochar, fruit quality, sustainable agriculture, biochemical attributes

## Abstract

**Introduction:**

Sustainable nutrient management is essential for improving guava fruit quality and reducing reliance on chemical fertilizers.

**Methods:**

A field experiment was conducted during 2022–2024 at SKUAST-Jammu using a Randomized Block Design with 16 treatments and 3 replications. Organic inputs (vermicompost, biochar, jaggery, etc.) were evaluated.

**Results:**

Treatment T6 (VC 5 kg + BC 7.5 kg + JG 1.25 kg/tree) recorded highest TSS (13.72°Brix), total sugars (10.10%), Vitamin C (175.27 mg/100 g), and lowest acidity (0.24%).

**Discussion:**

Integration of vermicompost, biochar, and jaggery significantly improves fruit quality and supports sustainable guava production.

## 1 Introduction

Guava (*Psidium guajava* L.), a prominent fruit crop of the Myrtaceae family, is widely cultivated in tropical and subtropical regions of India and originated from tropical America, spanning Mexico to Peru (Kumar et al., 2020). In India, where it has been grown since the 17th century, guava ranks fourth in area and production after mango, banana, and citrus (Kadam et al., 2012; Saba et al., 2018). Its cultivation is concentrated in states like Uttar Pradesh, Maharashtra, and Bihar, with Uttar Pradesh, especially Allahabad being renowned for premium-quality guava. In Jammu & Kashmir UT, guava is extensively cultivated in districts such as Jammu, Samba, and Udhampur, where favorable agro-climatic conditions and well-drained loamy soils support high-quality production (Anonymous, 2021, 2024). Often referred to as the "Apple of the Tropics," guava is appreciated not just for its affordability and hardiness but for its remarkable nutritional and therapeutic qualities. The fruit is a rich source of vitamin C (210–305 mg/100 g), pectin (0.5–1.8%), fiber, and antioxidants, all of which contribute to improved shelf-life, taste, and health value (Bose and Mitra, 2001).

The quality of guava fruit including attributes such as nutritional content, flavor, firmness, size, and shelf life is significantly influenced by soil health and input management. Excessive and prolonged use of chemical fertilizers in guava orchards has led to nutrient imbalances, soil degradation, and residual toxicity, ultimately deteriorating fruit quality and posing risks to human health (Shanker et al., 2002; Alavanja et al., 2013; Manisalidis et al., 2020). Residual pesticides and heavy metals in fruit reduce consumer safety and compromise flavor, texture, and nutritional composition. Conversely, the growing awareness about these risks is driving a transition toward organic and natural farming, where sustainable soil amendments such as vermicompost, biochar, neem cake, poultry manure, cow urine, and farmyard manure are gaining prominence (Gahukar, 2007; Ponmozhi et al., 2019; Adama et al., 2022). These inputs enrich soil organic matter, enhance microbial diversity, and improve nutrient availability (N, P, K, Ca, Mg, S), thereby ensuring balanced nutrition throughout fruit development. For example, vermicompost and biochar improve soil structure, water retention, and nutrient-use efficiency, leading to larger, juicier, and more nutritious fruits. Neem cake and cow urine offer added benefits by controlling soil-borne pests naturally, which supports disease-free fruiting and improved external and internal fruit quality (Hatfield and Walthall, 2015; Kavitha et al., 2018).

Moreover, the integration of organic sources enhances the organoleptic properties of guava such as taste, aroma, and pulp texture, while also extending shelf life due to higher antioxidant content and lower microbial decay. This not only benefits consumer health but also increases market value and export potential, especially as global demand for organic and chemical-free fruits continues to rise (Chandel et al., 2017; Kamboj et al., 2023). Natural farming practices, rooted in Indian traditions and supported by ecological principles, provide a holistic approach to guava production. These systems emphasize the complete avoidance of synthetic inputs such as chemical fertilizers, pesticides, and other artificial agrochemicals. Instead, they promote biomass recycling and the utilization of on-farm resources like cow dung–urine preparations, compost, and mulching. Such practices not only reduce dependence on external inputs but also sustain soil health, enhance microbial activity, and improve fruit quality over time (Shyam et al., 2019; Kumar et al., 2023).

In the face of growing climate uncertainties and rising health awareness, the integration of organic amendments with sustainable farming practices offers a promising pathway for producing premium-quality guava fruits. Such an approach not only minimizes chemical residues but also enhances the fruit nutritional value, flavor, and safety, aligning perfectly with modern consumer preferences for healthy and eco-friendly produce. This approach not only enhances the sustainability of guava cultivation but also supports long-term soil fertility, ecological resilience, and farmer profitability (Babalad and Navali, 2021; Laishram et al., 2022). Thus, optimizing organic nutrient combinations tailored to regional conditions is key for producing superior guava cultivars like L-49 while maintaining environmental and economic sustainability.

## 2 Materials and methods

The present investigation was carried out at the orchard of Division of Fruit Science, Chatha campus, Sher-e-Kashmir University of Agricultural Sciences & Technology of Jammu (J&K) during the years 2022–23 and 2023-24.

### 2.1 Climate and weather conditions

**Figure 1:**
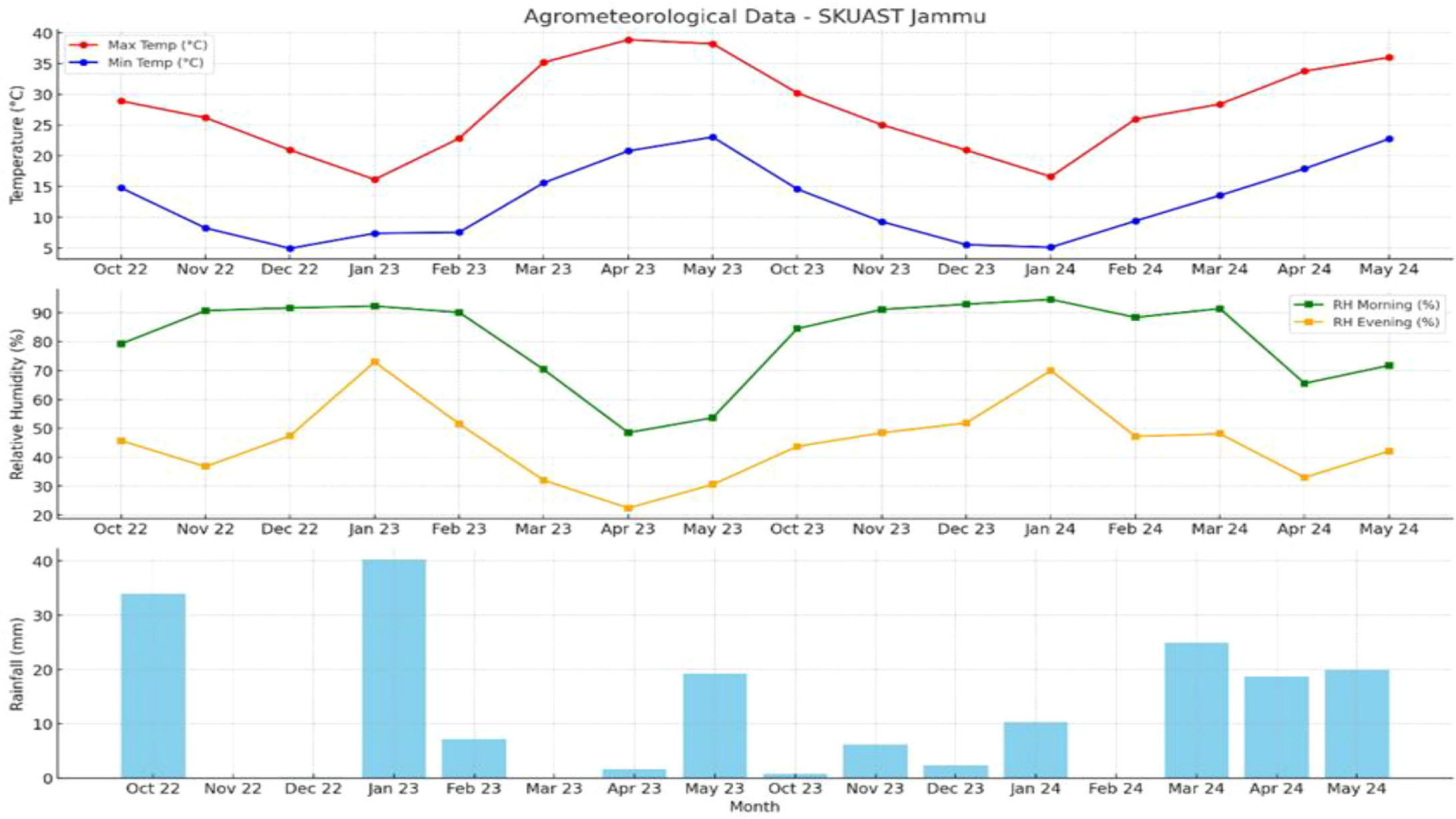
Agrometeorological characterization of the research farm at SKUAST-Jammu, J&K, India: a two-year study (October 2022 – May 2024).

The experimental site is located in the subtropical region at 32.43° N latitude and 74.7823° E longitude, with an elevation of 300 meters above sea level. The area receives an average annual rainfall of approximately 1200 mm, with nearly 70% occurring between July and October. The average annual maximum and minimum temperatures are 29.6°C and 16.7°C, respectively. Summers are typically hot, with temperatures ranging from 23.5°C to 40.5°C and relative humidity between 53.0% and 73.5%. Winters are relatively mild, with temperatures varying from 6.5°C to 21.7°C. The coldest month is January, when temperatures can drop to around 4°C, while the hottest temperatures, reaching up to 45°C, are usually recorded in June. Meteorological data relevant to the study were obtained from the nearby observatory and are detailed in Appendix-I and **Figure 1**.

### 2.2 Soil of the experimental plot

Soil samples were collected from various locations within the experimental orchard using a soil auger, covering a depth of 0–90 cm. The collected samples were thoroughly mixed to form a composite sample, which was then air-dried and ground into a fine powder for further analysis. The initial physico-chemical characteristics of the orchard soil were recorded and are presented in **Table 1**.

**Table 1:** Physio-chemical characteristics of the soil experimental site.

Particulars	Contents	Method used for analysis
A. Physical analysis		
Sand (%)	64.42	International Dispersion Method (Piper, 1950)
Silt (%)	18.84	International Dispersion Method (Piper, 1950)
Clay (%)	16.74	International Dispersion Method (Piper, 1950)
B. Chemical analysis		
Ph	6.78	1:2 soil:water suspension (Jackson, 1973)
Electrical conductivity (ds / m)	0.09	1:2 soil:water suspension (Jackson, 1973)
Organic Carbon (%)	0.42	Wet Oxidation Method (Walkley and Black, 1934)
Available Nitrogen (kg N / ha)	203.24	Alkaline Permanganate Method (Subbiah and Asija, 1956)
Available Phosphorus (kg P_2_O_5_ / ha)	12.53	Olsen’s Method (Olsen et al., 1954)
Available Potassium (kg K_2_O / ha)	138.65	Flame Photometer (Black, 1965)
Available Calcium (meq / 100 g)	10.56	Versenate Titration Method (Mervin and Peech, 1951)
Available Magnesium (meq / 100 g)	2.32	Versenate Titration Method Mervin and Peech, 1951)

The soil at the SKUAST-Jammu research farm is a sandy loam with a near-neutral pH (6.78), low organic carbon (0.42), and a fertility profile characterized by low available nitrogen and phosphorus but medium potassium levels.

According to the data in **Table 1**, the soil exhibited a sandy loam texture and ranged from acidic to neutral in pH. The available nitrogen content was found to be low, whereas the levels of available phosphorus and potassium were within the medium range.

### 2.3 Preparation and source of organic materials utilized in the treatments

**Table:** 

Material	Preparation / source
Vermicompost	Prepared from well-decomposed cow dung and agro-residues (vegetable/crop wastes) using *Eisenia fetida* earthworms under standard vermicomposting conditions.
Cow Urine	Freshly collected from indigenous cows and stored in airtight containers before application.
Neem Cake	By-product obtained after oil extraction from neem (*Azadirachta indica*) seeds; procured from local oil mills.
Biochar	Produced by pyrolysis of crop residues (e.g., rice husk, maize cobs) under limited oxygen supply at 350–450°C.
Jaggery	Locally procured concentrated product of sugarcane juice, rich in sucrose and micronutrients.
Poultry Manure	Collected from poultry farms; sun-dried and partially decomposed before application.
Farmyard Manure (FYM)	Prepared from the dung and urine of cattle mixed with bedding material and crop residues, composted for 3–4 months.

*Description related to preparation and source of organic materials prepared at laboratory of Division of Fruit Science, SKUAST-Jammu, INDIA.

### 2.4 Chemical composition of various organic materials utilized in the treatments

**Table:** 

Material	N (%)	P (%)	K (%)	Other nutrients
Vermicompost	1.6 - 2.0	0.5 - 1.0	0.5 - 0.9	Calcium (Ca): 1.0-2.0%, Magnesium (Mg): 0.5-1.0%
Cow Urine	0.5 - 1.0	0.02 - 0.10	0.15 - 0.30	Sulfur (S): 0.03-0.05%, Urea content
Neem Cake	2.0 - 5.0	0.5 - 1.0	1.0 - 1.5	Azadirachtin: 0.2-0.5%, Organic Carbon: 15-20%
Biochar	0.5 - 1.0	0.1 - 0.5	1.0 - 2.0	Carbon (C): 70-80%, pH: 7.5-8.5
Jaggery	–	–	–	Rich in Carbohydrates (Sucrose), Minor minerals: Fe, Ca
Poultry Manure	1.5 - 2.5	1.0 - 1.5	0.5 - 1.0	Organic Matter: 30-40%, Ca, Mg, S
Farmyard Manure	0.5 - 1.5	0.2 - 0.5	0.5 - 1.5	Organic Carbon: 15-25%, Calcium (Ca), Magnesium (Mg)

### 2.5 Treatment details

The experiment consisted of sixteen treatments designed to evaluate the effects of various organic nutrient management combinations on guava cv. L-49. The treatments included: T_1_ – vermicompost at 15.00 kg/tree as a 100% replacement of nitrogen (N) through vermicompost; T_2_ – vermicompost at 10.00 kg/tree combined with cow urine at 0.50 liter/tree and neem cake at 1.00 kg/tree; T_3_ – vermicompost at 10.00 kg/tree with cow urine at 1.00 liter/tree and neem cake at 1.50 kg/tree; T_4_ – vermicompost at 10.00 kg/tree with cow urine at 1.50 liter/tree and neem cake at 2.00 kg/tree; T_5_ – vermicompost at 5.00 kg/tree, biochar at 5.00 kg/tree, and jaggery at 1.00 kg/tree; T_6_ – vermicompost at 5.00 kg/tree, biochar at 7.50 kg/tree, and jaggery at 1.25 kg/tree; T_7_ – vermicompost at 5.00 kg/tree, biochar at 10.00 kg/tree, and jaggery at 1.50 kg/tree; T_8_ – poultry manure at 5.00 kg/tree as a 100% replacement of N through poultry manure; T_9_ – poultry manure at 3.00 kg/tree with biochar at 4.00 kg/tree and jaggery at 0.50 kg/tree; T_10_ – poultry manure at 3.00 kg/tree with biochar at 5.00 kg/tree and jaggery at 0.75 kg/tree; T_11_ – poultry manure at 3.00 kg/tree with biochar at 6.00 kg/tree and jaggery at 1.00 kg/tree; T_12_ – farmyard manure at 15.00 kg/tree as a 100% replacement of N through farmyard manure; T_13_ – farmyard manure at 10.00 kg/tree with cow urine at 0.75 liter/tree and neem cake at 1.50 kg/tree; T_14_ – farmyard manure at 10.00 kg/tree with cow urine at 1.25 liter/tree and neem cake at 1.75 kg/tree; T_15_ – farmyard manure at 10.00 kg/tree with cow urine at 1.75 liter/tree and neem cake at 2.00 kg/tree; and T_16_ – the recommended Package of Practices (control).

#### 2.5.1 Treatment nomenclature

The treatments applied in the experiment, along with their corresponding short names, are detailed in the table below. These treatments involve various combinations of organic and inorganic nutrient sources applied to trees, with quantities specified per tree as shown in **Table 2**.

**Table 2:** Nomenclature of treatments applied to trees.

Treatment code	Treatment details
T_1_	VC (15 kg/tree) (100% replacement of N through VC)
T_2_	VC (10 kg/tree) + CU (0.5 liter/tree) + NC (1.0 kg/tree)
T_3_	VC (10 kg/tree) + CU (1.0 liter/tree) + NC (1.5 kg/tree)
T_4_	VC (10 kg/tree) + CU (1.5 kg/tree) + NC (2.0 kg/tree)
T_5_	VC (5 kg/tree) + BC (5.0 kg/tree) + JG (1.0 kg/tree)
T_6_	VC (5 kg/tree) + BC (7.5 kg/tree) + JG (1.25 kg/tree)
T_7_	VC (5 kg/tree) + BC (10 kg/tree) + JG (1.50 kg/tree)
T_8_	PM (5 kg/tree) (100% replacement of N through PM)
T_9_	PM (3 kg/tree) + BC (4.0 kg/tree) + JG (0.5 kg/tree)
T_10_	PM (3 kg/tree) + BC (5.0 kg/tree) + JG (0.75 kg/tree)
T_11_	PM (3 kg/tree) + BC (6.0 kg/tree) + JG (1.0 kg/tree)
T_12_	FM (15 kg/tree) (100% replacement of N through FYM)
T_13_	FM (10 kg/tree) + CU (0.75 liter/tree) + NC (1.5 kg/tree)
T_14_	FM (10 kg/tree) + CU (1.25 liter/tree) + NC (1.75 kg/tree)
T_15_	FM (10 kg/tree) + CU (1.75 liter/tree) + NC (2.0 kg/tree)
T_16_	Control

### 2.6 Experiment details

The field experiment was conducted on guava cv. L-49 using a Randomized Block Design (RBD) with 16 treatments and three replications. Each replication comprised 16 trees, one for each treatment, making a total of 48 trees. The orchard was planted at a spacing of 3 m × 3 m, resulting in three rows (replications) each containing 16 trees in a line. The distribution of treatments within each replication was randomized independently. The schematic layout is given in **Table 3**.

**Table 3:** Schematic layout of experimental site (3 rows × 16 trees per row).

Replication/block	P1	P2	P3	P4	P5	P6	P7	P8	P9	P10	P11	P12	P13	P14	P15	P16
Replication 1/Block 1(North Row )	T_4_	T_9_	T_1_	T_15_	T_8_	T_12_	T_6_	T_14_	T_11_	T_3_	T_10_	T_16_	T_13_	T_2_	T_7_	T_5_
Replication 2/Block 2 (Central Row )	T_7_	T_2_	T_10_	T_5_	T_12_	T_1_	T_16_	T_3_	T_14_	T_9_	T_4_	T_8_	T_11_	T_6_	T_13_	T_15_
Replication 3/Block 3 (South Row )	T_11_	T_6_	T_15_	T_12_	T_2_	T_9_	T_8_	T_5_	T_1_	T_7_	T_14_	T_4_	T_13_	T_3_	T_16_	T_10_

P1–P16 represent sequential experimental positions within each replication. Treatments were randomly allocated within each block following the principles of Randomized Block Design (RBD).

(Treatment order within each replication was randomized; table shows a representative arrangement.).

Neighboring effects were minimized as each replication was planted in a separate row, thereby reducing interaction across replications, while randomization within each row ensured an unbiased distribution of treatments. A surrounding guard row of guava trees protected the experimental plots from border effects, and 3 m wide inter-row alleys further limited shading, root overlap, and nutrient competition. Moreover, since each treatment was represented by a single tree per replication, the likelihood of direct treatment-to-treatment interference was negligible.

### 2.7 Plant material identification and voucher specimen

The guava plants (*Psidium guajava* L.) cultivar L-49 utilized in this study were officially recognized by Dr. A.K. Singh, Principal Scientist, Division of Fruits, ICAR–Central Institute for Subtropical Horticulture (CISH), Lucknow, India. A voucher specimen was created according to conventional herbarium methods, specifically pressed and dried with collection metadata, and placed in the Herbarium of ICAR-CISH, Lucknow, India, for future reference. The voucher specimen received the accession number CISH/FRU/2025/049. This approach adheres to established best practices: voucher specimens must be deposited in accredited public herbaria to facilitate verification, repeatability, and long-term botanical documentation. Additionally, ICAR-CISH is esteemed for its contributions to subtropical horticulture research, particularly in guava germplasm, and operates nursery and herbarium facilities to facilitate research and germplasm documentation.

### 2.8 Methodology of the experiment

**Figure 2:**
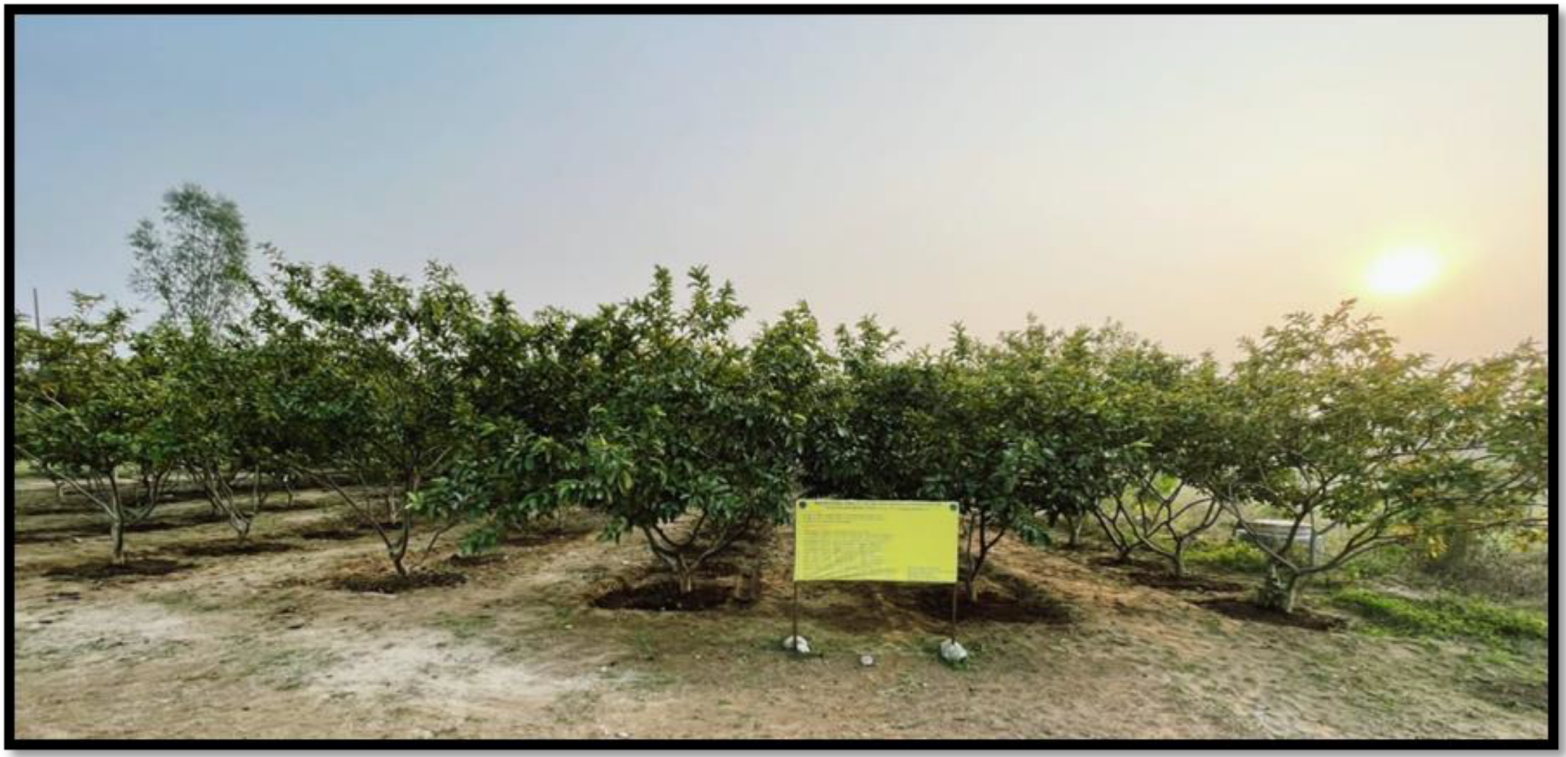
General view of experimental site at Research Farm, SKUAST-Jammu, J&K, INDIA.

**Figure 3:**
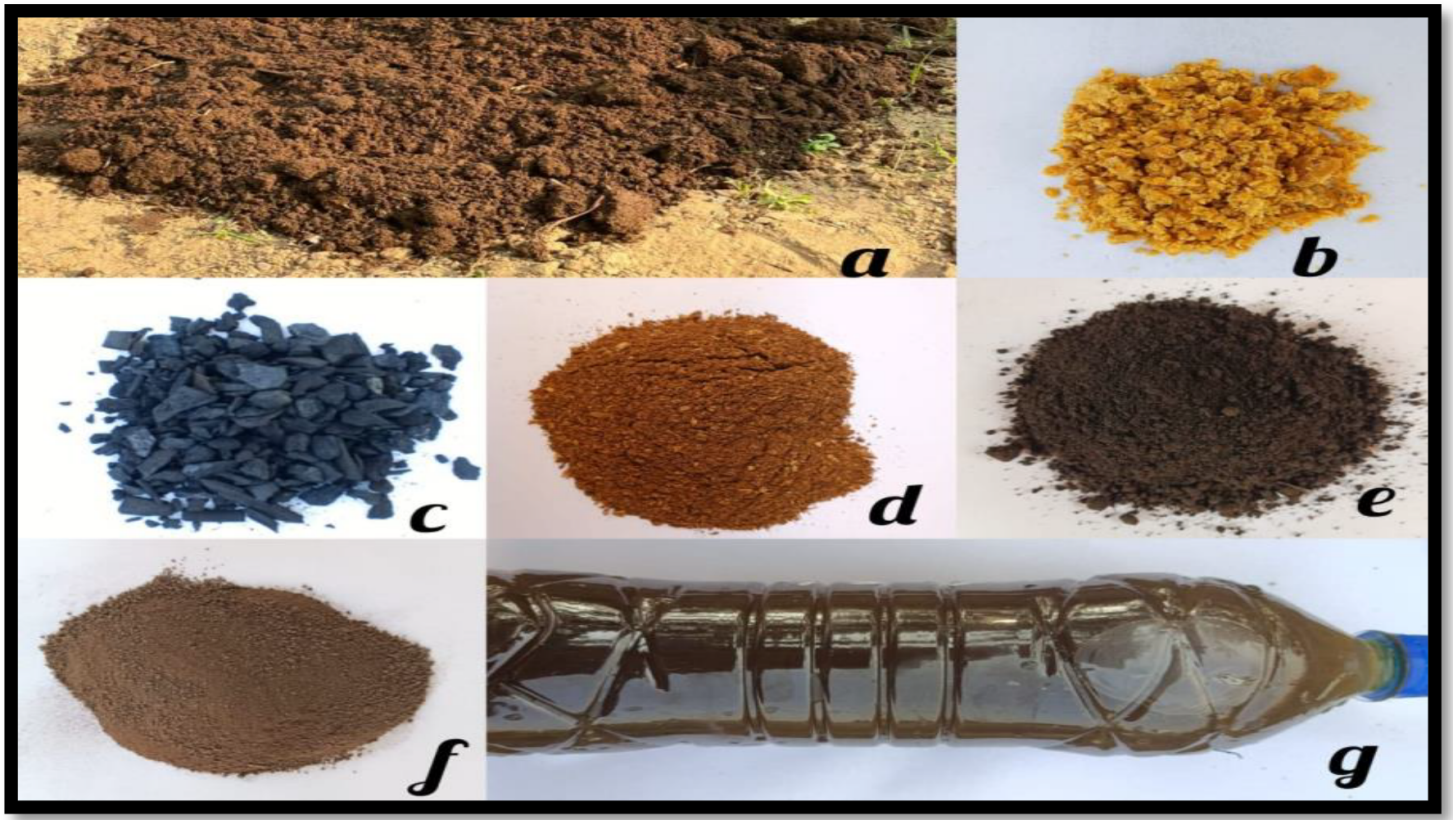
Organic nutrient sources applied in this experiment where, panel **(a)** represents farmyard manure, panel **(b)** represents jaggery, panel **(c)** represents biochar, panel **(d)** represents neem cake, panel **(e)** represents vermicompost, panel **(f)** represents poultry manure and panel **(g)** represents cow urine.

**Figure 4:**
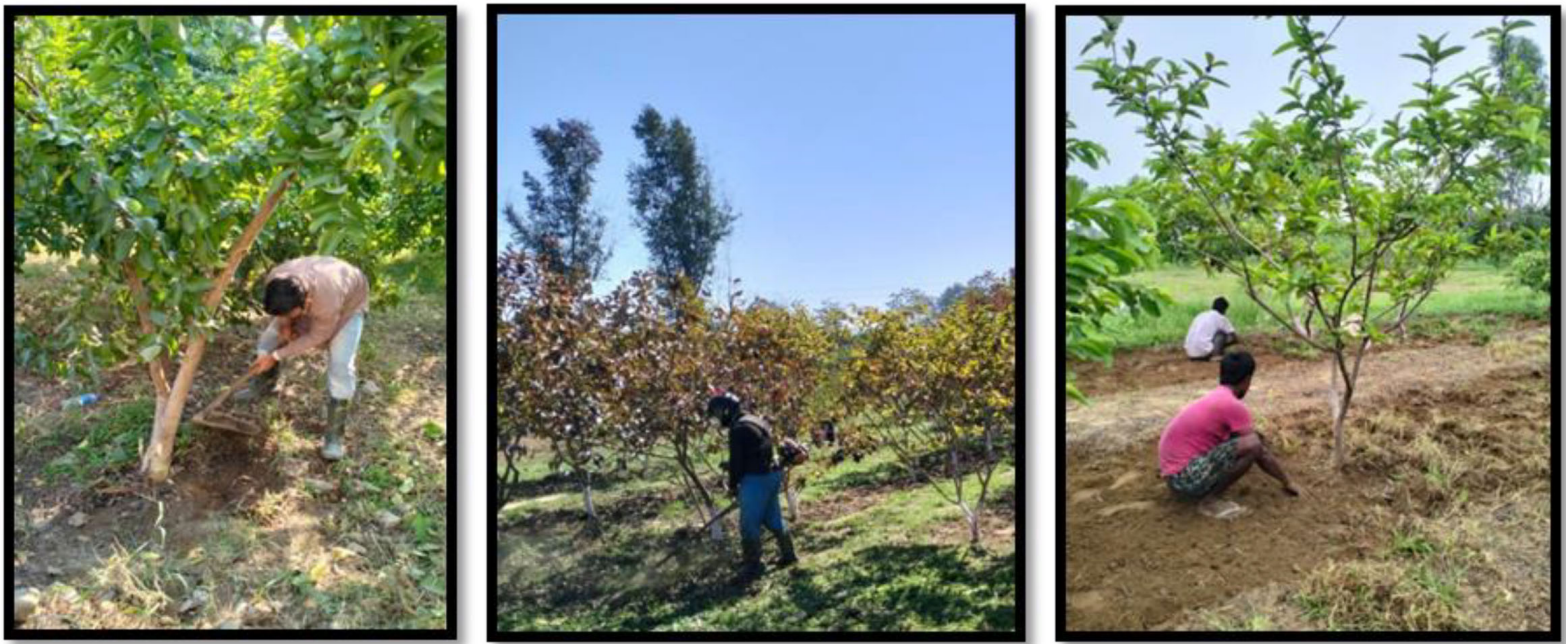
Weeding operations conducted during both seasons using manual labor and bush cutters to control weed growth.

**Figure 5:**
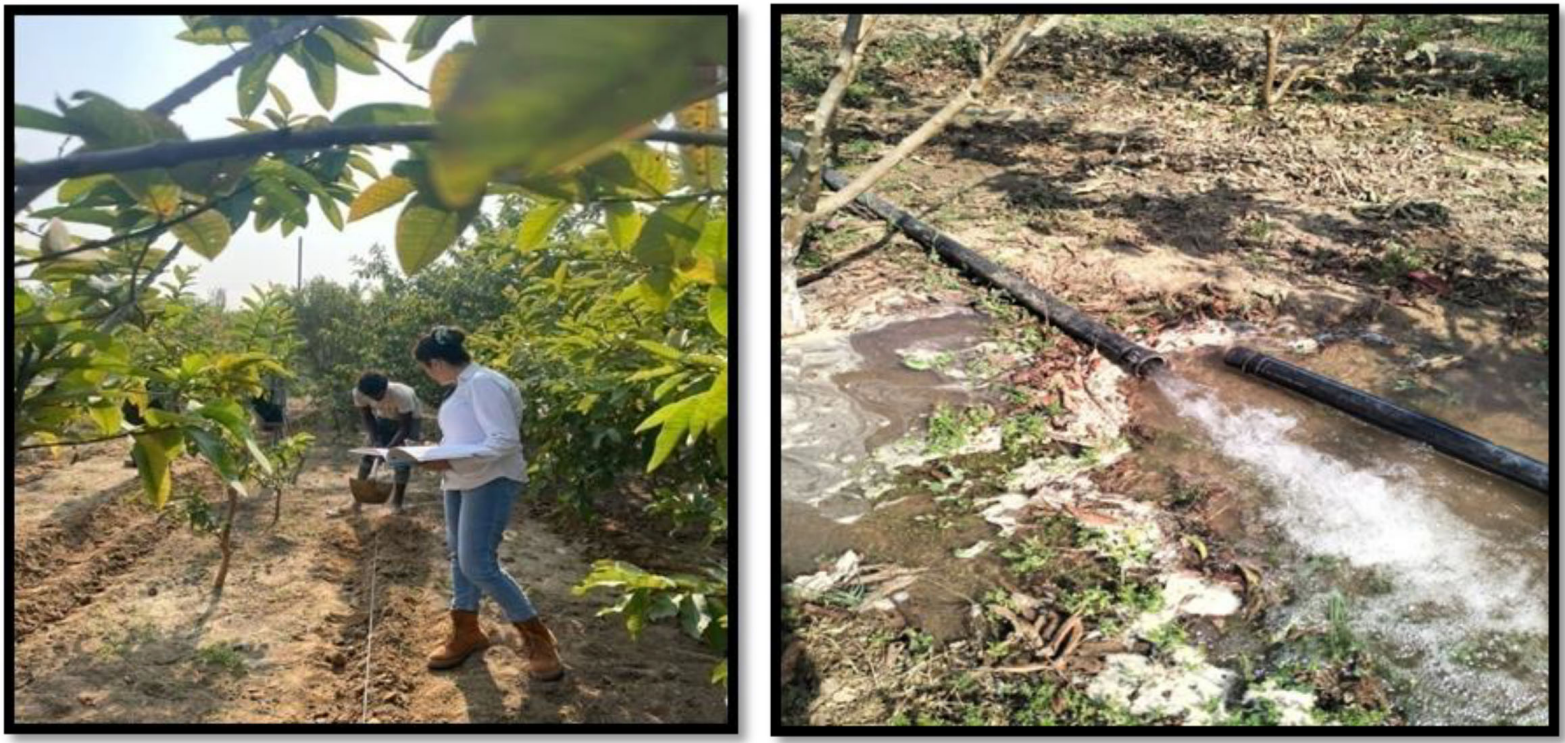
Irrigation system in operation during both seasons to ensure consistent water supply for optimal crop growth and yield.

Six-year-old guava plants (cv. L-49) having uniform vigor, size, and productivity and planted at a spacing of 3 m × 3 m were selected from the research farm of SKUAST-Jammu for conducting the study. The treatments were applied to evaluate their effect on fruit quality and productivity of guava during the experimental period. Organic amendments were initially incorporated in November, aligning with the guava growth cycle to ensure optimal nutrient uptake during the winter months. Subsequently, the treatment were applied at three- month intervals during both years of the study, considering the slow decomposition nature of organic materials. Vermicompost, poultry manure and farmyard manure were evenly distributed around the base of each guava tree and lightly tilled into the soil for efficient integration. Biochar was directly mixed into the soil to enhance its physical and chemical properties, improving soil health. Jaggery was dissolved in water to create a liquid fertilizer, which was applied to the soil to boost nutrient absorption and efficacy. In specific treatments, cow urine was combined with vermicompost and neem cake and infused into the soil to enhance nutrient availability and stimulate microbial activity. The treatment doses were standardized on an equivalent nutrient basis in relation to recommended dose of nutrients and ensured that the observed differences in fruit quality parameters were primarily associated with the nature and function properties of organic materials rather than disparities in total nutrient input. This meticulous application strategy was carefully designed to maximize the efficacy of organic amendments, supporting the tree nutrient needs and fostering improved fruit quality and yield. The control treatment in this context refers to following the recommended package of practices without any additional or experimental treatments. This involves strictly adhering to the specified quantities of Urea (700 g), Di-Ammonium Phosphate (275 g), and Muriate of Potash (135 g) according to the tree’s age, along with the recommended timing of application. This standard practice serves as a baseline against which other treatments can be compared. All other orchard management practices, including irrigation, pruning, plant protection measures, and intercultural operations, were kept uniform across all treatments throughout the experimental period to ensure that the observed differences were primarily due to treatment effects. The evaluation of guava fruit quality involved several biochemical estimations following standard protocols. Total Soluble Solids (TSS) were measured in°Brix using a hand refractometer, with temperature corrections standardized at 20°C. Total sugars were estimated by hydrolyzing 25 g of pulp, followed by titration with Fehling's solution after clarification using lead acetate and potassium oxalate, in line with (AOAC, 1995). Reducing sugars were assessed from the unhydrolyzed aliquot using the same titration method, and non-reducing sugars were calculated by subtracting reducing sugars from total sugars and multiplying by 0.95. Titratable acidity was determined by titrating pulp extract with 0.1 N NaOH using phenolphthalein, with results expressed as citric acid equivalent as per (AOAC, 2002). Vitamin C content was quantified using the 2,6-dichlorophenol indophenol dye titration method involving dye standardization, pulp extraction in metaphosphoric acid, and titration to a persistent pink endpoint (**Figures 2**–**5**). Lastly, pectin content was estimated following (Ranganna, 1979), wherein calcium pectate was precipitated from hot water extracts of 25 g macerated fruit, and quantified gravimetrically after treatment with NaOH, acetic acid, and calcium chloride, providing a comprehensive profile of fruit biochemical quality.

### 2.9 Description of experimental setup

**Table:** 

Aspect	Description
Plant selection	Six-year-old guava plants of uniform vigor, size, and productivity were selected at the Research Farm, SKUAST-Jammu.
Treatment implementation	Treatments assessed impact on guava fruit quality and productivity. Organic amendments applied in November to align with the guava growth cycle for optimal nutrient uptake during winter months.
Organic amendments application	Vermicompost, poultry manure, and farmyard manure evenly distributed around tree base and lightly tilled into soil. Biochar mixed directly into soil to enhance physical and chemical properties. Jaggery dissolved in water as liquid fertilizer applied to soil. Cow urine combined with vermicompost and neem cake, infused into soil to enhance nutrient availability and microbial activity.
Control treatment	Recommended package of practices: Urea (700 g), Di-Ammonium Phosphate (275 g), and Muriate of Potash (135 g) per tree, based on age, with standard application timing.

### 2.10 Biochemical estimation methods for guava fruit quality assessment

**Table:** 

Biochemical estimations of parameters	Methodology used
Total soluble solids (TSS):	Measured in °Brix using hand refractometer, standardized at 20°C.
Total sugars:	25 g pulp hydrolyzed, clarified with lead acetate & potassium oxalate, titrated with Fehling’s solution [65].
Reducing sugars:	Determined from unhydrolyzed aliquot using Fehling’s solution titration.
Non-reducing sugars:	Calculated as (Total sugars – Reducing sugars) × 0.95.
Titratable acidity:	Pulp extract titrated with 0.1 N NaOH using phenolphthalein; expressed as citric acid equivalent [66].
Vitamin C:	Estimated by titration with 2,6-dichlorophenol indophenol dye using pulp extracted in metaphosphoric acid.
Pectin content:	Calcium pectate precipitated from hot water extract of 25 g macerated fruit; quantified gravimetrically after NaOH, acetic acid, and CaCl_2_ treatment [67].

### 2.11 Data collection and analysis

The experimental data recorded during both seasons (2022–2023 and 2023–2024) were subjected to analysis of variance (ANOVA) using a Randomized Block Design using R software (version 4.2.3) with the help of the ‘agricolae’ package as described by Gomez and Gomez (1984). Data were analyzed separately for each year and also subjected to pooled analysis across years. In the pooled analysis, year was treated as an additional factor to test the effect of year and treatment × year interaction. The significance of differences among treatment means was tested using the Least Significant Difference (LSD) test at P ≤ 0.05. The assumptions of ANOVA, including normality of residuals and homogeneity of variances, were verified prior to analysis.

## 3 Results

### 3.1 Total soluble solids (°Brix)

The data with respect to total soluble solids (%) presented in **Table 4** showed the synergistic effect of different organic nutrient sources on total soluble solids (˚Brix) of guava and was investigated over two years, 2022–2023 and 2023-2024. During the year 2022-2023, the significant increase in TSS was recorded in T_6_ (13.57 ˚Brix), statistically at par with treatment T_7_ (13.04 ˚Brix) and was followed by treatment T_5_ (12.66 ˚Brix) as compared to control T_16_ (9.38 ˚Brix). In contrast, treatment T_8_ (9.27 ˚Brix) had lowest TSS value. In the year 2023-2024, the trends remained consistent, with T_6_ (13.87 ˚Brix) again showed the significant highest TSS, statistically equivalent with treatments T_7_ (13.58 ˚Brix) and T_5_ (13.05 ˚Brix) over control T_16_ (10.69 ˚Brix). The treatment T_8_, which had the lowest TSS (9.47 ˚Brix), consistently showed lower performance in both the years. When the results from both years were consolidated, the treatment T_6_ (13.72 ˚Brix) that showed the significant highest average fruit TSS, statistically at par with treatment T_7_ (13.31 ˚Brix) as compared to control T_16_ (10.04 ˚Brix). The pooled results also revealed that treatment T_8_ (9.37 ˚Brix) showed the lowest value in TSS. These results indicate that although T_6_ recorded comparatively higher TSS values, treatment T_7_ showed statistically similar performance, suggesting that both treatments were comparable in their effect on fruit soluble solids.

**Table 4:** Effect of organic sources of nutrients on total soluble solids (˚Brix) of guava cv. L-49.

Treatments		Grouping (α = 0.05)	Total soluble solids (^0^Brix)		
			2022–2023	2023–2024	Pooled
T_1_	VC (15 kg/tree) (100% replacement of N through VC)	Efg	9.61	10.95	10.28
T_2_	VC (10 kg/tree) + CU (0.5 liter/tree) + NC (1.0 kg/tree)	Def	10.62	10.88	10.75
T_3_	VC (10 kg/tree) + CU (1.0 liter/tree) + NC (1.5 kg/tree)	Cdef	11.03	11.25	11.14
T_4_	VC (10 kg/tree) + CU (1.5 kg/tree) + NC (2.0 kg/tree)	Bc	12.03	11.87	11.95
T_5_	VC (5 kg/tree) + BC (5.0 kg/tree) + JG (1.0 kg/tree)	Ab	12.66	13.05	12.86
T_6_	VC (5 kg/tree) + BC (7.5 kg/tree) + JG (1.25 kg/tree)	A	13.57	13.87	13.72
T_7_	VC (5 kg/tree) + BC (10 kg/tree) + JG (1.50 kg/tree)	A	13.04	13.58	13.31
T_8_	PM (5 kg/tree) (100% replacement of N through PM)	G	9.27	9.47	9.37
T_9_	PM (3 kg/tree) + BC (4.0 kg/tree) + JG (0.5 kg/tree)	Bcd	11.56	12.07	11.82
T_10_	PM (3 kg/tree) + BC (5.0 kg/tree) + JG (0.75 kg/tree)	Cde	11.27	11.48	11.38
T_11_	PM (3 kg/tree) + BC (6.0 kg/tree) + JG (1.0 kg/tree)	Bc	11.81	12.04	11.92
T_12_	FM (15 kg/tree) (100% replacement of N through FYM)	cdef	10.82	10.89	10.85
T_13_	FM (10 kg/tree) + CU (0.75 liter/tree) + NC (1.5 kg/tree)	cdef	10.39	11.37	10.88
T_14_	FM (10 kg/tree) + CU (1.25 liter/tree) + NC (1.75 kg/tree)	Ef	10.36	10.68	10.52
T_15_	FM (10 kg/tree) + CU (1.75 liter/tree) + NC (2.0 kg/tree)	cdef	10.54	11.38	10.96
T_16_	Recommended POP (Control)	Fg	9.38	10.69	10.04
S.E.m (±)			0.48	0.66	0.98
*CD (0.05)*			1.39	1.91	1.14

*CD(0.05)*, Critical Difference at 5%; S.E.m(±), Standard error of mean.

**Table:** Analysis of variance for TSS (°Brix).

Source of variation	Df	Sum of squares	Mean square	F value	P value
Treatment	15	126.896	8.4597	8.591	<0.001 ***
Replication	2	0.130	0.0652	0.066	0.936
Environment (Year)	1	5.349	5.3487	5.432	0.023 *
Treatment × Environment	15	4.183	0.2789	0.283	0.995
Error	62	61.051	0.9847	—	—
Total	95	197.609	—	—	—

*→ Significant at P ≤ 0.05, *** → Highly significant at P ≤ 0.001.

### 3.2 Total sugars (%)

From the perusal of the data presented in **Table 5**, the total sugars (%) was significantly affected by the application of organic sources of nutrients during 2022–2023 and 2023-2024, with pooled data provided a comprehensive overview of the treatment effects. In the year 2022-2023, the significant highest total sugars was observed in T_6_ (9.82%), *followed by T_5_ (9.73*%*)* as compared to control T_16_ (6.76%) and the lowest total sugars was noted in treatment T_12_ (5.23%). The trends in 2023–2024 largely mirrored those of the previous year. T_6_ (10.38%) again achieved the significant highest total sugars, followed by treatment T_7_ (9.08%), as compared to control T_16_ (8.04%), while T_12_ (5.17%) remained the lowest-performing treatment. When the results from both years were consolidated, T_6_ (10.10%) showed the significant highest value, succeeded by treatment T_5_ (8.97%), while control T_16_ (5.20%) depicted the lowest value.

**Table 5:** Effect of organic sources of nutrients on total sugars (%) of guava cv. L-49.

Treatments		Grouping (α = 0.05)	Total sugars (%)		
			2022-2023	2023-2024	Pooled
T_1_	VC (15 kg/tree) (100% replacement of N through VC)	e	7.51	8.84	8.18
T_2_	VC (10 kg/tree) + CU (0.5 liter/tree) + NC (1.0 kg/tree)	d	7.76	8.92	8.34
T_3_	VC (10 kg/tree) + CU (1.0 liter/tree) + NC (1.5 kg/tree)	d	8.03	8.65	8.34
T_4_	VC (10 kg/tree) + CU (1.5 kg/tree) + NC (2.0 kg/tree)	g	8.39	7.68	8.04
T_5_	VC (5 kg/tree) + BC (5.0 kg/tree) + JG (1.0 kg/tree)	b	9.73	8.21	8.97
T_6_	VC (5 kg/tree) + BC (7.5 kg/tree) + JG (1.25 kg/tree)	a	9.82	10.38	10.10
T_7_	VC (5 kg/tree) + BC (10 kg/tree) + JG (1.50 kg/tree)	c	8.54	9.08	8.81
T_8_	PM (5 kg/tree) (100% replacement of N through PM)	i	7.23	8.45	7.84
T_9_	PM (3 kg/tree) + BC (4.0 kg/tree) + JG (0.5 kg/tree)	f	7.63	8.53	8.08
T_10_	PM (3 kg/tree) + BC (5.0 kg/tree) + JG (0.75 kg/tree)	j	6.66	8.77	7.72
T_11_	PM (3 kg/tree) + BC (6.0 kg/tree) + JG (1.0 kg/tree)	h	7.11	8.65	7.88
T_12_	FM (15 kg/tree) (100% replacement of N through FYM)	o	5.23	5.17	5.20
T_13_	FM (10 kg/tree) + CU (0.75 liter/tree) + NC (1.5 kg/tree)	m	7.05	7.63	7.34
T_14_	FM (10 kg/tree) + CU (1.25 liter/tree) + NC (1.75 kg/tree)	k	7.02	8.32	7.67
T_15_	FM (10 kg/tree) + CU (1.75 liter/tree) + NC (2.0 kg/tree)	n	6.46	7.56	7.01
T_16_	Recommended POP (Control)	l	6.76	8.04	7.40
S.E.m (±)			0.01	0.01	0.01
*CD (0.05)*			0.04	0.04	0.03

*CD(0.05)*, Critical Difference at 5%, S.E.m(±): Standard error of mean.

**Table:** Analysis of variance for total sugars (%).

Source of Variation	df	Sum of squares	Mean square	F value	P value
Treatment	15	96.380	6.4253	12848.05	<0.001 ***
Replication	2	0.000	0.0001	0.13	0.8809 ns
Environment (Year)	1	13.395	13.3952	26785.01	<0.001 ***
Treatment × Environment	15	17.989	1.1993	2398.06	<0.001 ***
Error	62	0.031	0.0005	—	—
Total	95	127.795	—	—	—

*→ Significant at P ≤ 0.05, ** → Significant at P ≤ 0.01, *** → Highly significant at P ≤ 0.001, ns → Non-significant

### 3.3 Reducing sugars (%)

The results presented in **Table 6** revealed that the application of organic sources of nutrients significantly improved the reducing sugars as compared to control at the time of harvest and was assessed over two growing seasons, 2022–2023 and 2023-2024, with pooled data providing a detailed overview of the treatment effects. During the year 2022-2023, the significant highest reducing sugars was recorded for treatment T_7_ (5.86%), *followed by treatments T_8_ (5.67*%*)* as compared to control T_16_ (5.09%) and lowest value was noted in treatment T_12_ (3.61%). In the subsequent year 2023-2024, the trends were largely similar with those assessed in the previous year, treatment T_6_ (6.43%) achieved the significant highest reducing sugars followed by treatment T_7_ (6.08%), in comparison with control T_16_ (5.04%). When the results from both years were consolidated, T_6_ (6.02%) demonstrated the most consistent and substantial effect on reducing sugars, followed by treatment T_7_ (5.97%) as compared to control T_16_ (5.06%). On the other hand, the treatment T_12_ (3.59%) showed lowest value in reducing sugars.

**Table 6:** Effect of organic sources of nutrients on reducing sugars (%) of guava cv. L-49.

Treatments		Grouping (α = 0.05)	Reducing sugars (%)		
			2022–2023	2023–2024	Pooled
T_1_	VC (15 kg/tree) (100% replacement of N through VC)	L	4.75	4.89	4.82
T_2_	VC (10 kg/tree) + CU (0.5 liter/tree) + NC (1.0 kg/tree)	K	4.81	5.17	4.99
T_3_	VC (10 kg/tree) + CU (1.0 liter/tree) + NC (1.5 kg/tree)	G	5.06	5.66	5.36
T_4_	VC (10 kg/tree) + CU (1.5 kg/tree) + NC (2.0 kg/tree)	F	5.11	5.77	5.44
T_5_	VC (5 kg/tree) + BC (5.0 kg/tree) + JG (1.0 kg/tree)	E	5.03	5.93	5.48
T_6_	VC (5 kg/tree) + BC (7.5 kg/tree) + JG (1.25 kg/tree)	A	5.61	6.43	6.02
T_7_	VC (5 kg/tree) + BC (10 kg/tree) + JG (1.50 kg/tree)	B	5.86	6.08	5.97
T_8_	PM (5 kg/tree) (100% replacement of N through PM)	C	5.67	5.76	5.72
T_9_	PM (3 kg/tree) + BC (4.0 kg/tree) + JG (0.5 kg/tree)	H	5.13	5.21	5.17
T_10_	PM (3 kg/tree) + BC (5.0 kg/tree) + JG (0.75 kg/tree)	D	5.19	6.05	5.62
T_11_	PM (3 kg/tree) + BC (6.0 kg/tree) + JG (1.0 kg/tree)	I	5.20	4.95	5.07
T_12_	FM (15 kg/tree) (100% replacement of N through FYM)	N	3.61	3.57	3.59
T_13_	FM (10 kg/tree) + CU (0.75 liter/tree) + NC (1.5 kg/tree)	M	4.78	4.41	4.59
T_14_	FM (10 kg/tree) + CU (1.25 liter/tree) + NC (1.75 kg/tree)	J	5.04	5.03	5.03
T_15_	FM (10 kg/tree) + CU (1.75 liter/tree) + NC (2.0 kg/tree)	I	5.29	5.57	5.43
T_16_	Recommended POP (Control)	I	5.09	5.04	5.06
S.E.m (±)			0.01	0.01	0.00
*CD (0.05)*			0.02	0.03	0.02

*CD (0.05)*, Critical Difference at 5%; S.E.m (±), Standard error of mean.

**Table:** Analysis of variance for reducing sugar (%).

Source of variation	df	Sum of squares	Mean square	F value	P value
Treatment	15	30.7657	2.05105	9967.20	<0.001 ***
Replication	2	0.0077	0.00385	18.73	<0.001 ***
Environment (Year)	1	1.7361	1.73613	8436.82	<0.001 ***
Treatment × Environment	15	3.5645	0.23763	1154.79	<0.001 ***
Error	62	0.0128	0.00021	—	—
Total	95	36.0868	—	—	—

*** → Highly significant at P ≤ 0.001.

### 3.4 Non-reducing sugars (%)

Data pertaining to non-reducing sugars (%) presented in **Table 7** clearly showed that the significant variation among treatments and was assessed across two growing seasons, 2022–2023 and 2023-2024, with pooled data provided a comprehensive overview of the treatment effects. In the year 2022-2023, the significant highest non-reducing sugars was observed in T_5_ (4.71%), *followed by T_6_ (4.15*%*)* as compared to control T_16_ (1.68%) and the lowest value in non-reducing sugars was found in treatment T_12_ (1.61%). The trends in 2023–2024 largely mirrored those of the previous year with treatment T_1_ (3.91%), achieved the significant highest non-reducing sugars, followed by treatment T_6_ (3.86%) as compared to control T_16_ (2.85%) while T_12_ (1.64%) remained the lowest-performing treatment. After combined the data from both years, treatment T_6_ (4.01%) showed the significant highest value in non-reducing sugars, followed by treatment T_5_ (3.49%) as compared to control T_16_ (2.26%).

**Table 7:** Effect of organic sources of nutrients on non-reducing sugars (%) of guava cv. L-49.

Treatments		Grouping (α = 0.05)	Non-reducing sugars (%)		
			2022–2023	2023–2024	Pooled
T_1_	VC (15 kg/tree) (100% replacement of N through VC)	C	2.76	3.91	3.33
T_2_	VC (10 kg/tree) + CU (0.5 liter/tree) + NC (1.0 kg/tree)	C	2.96	3.74	3.35
T_3_	VC (10 kg/tree) + CU (1.0 liter/tree) + NC (1.5 kg/tree)	D	2.94	2.99	2.97
T_4_	VC (10 kg/tree) + CU (1.5 kg/tree) + NC (2.0 kg/tree)	F	3.26	2.02	2.64
T_5_	VC (5 kg/tree) + BC (5.0 kg/tree) + JG (1.0 kg/tree)	B	4.71	2.27	3.49
T_6_	VC (5 kg/tree) + BC (7.5 kg/tree) + JG (1.25 kg/tree)	a	4.15	3.86	4.01
T_7_	VC (5 kg/tree) + BC (10 kg/tree) + JG (1.50 kg/tree)	de	2.66	3.08	2.87
T_8_	PM (5 kg/tree) (100% replacement of N through PM)	h	1.55	2.65	2.10
T_9_	PM (3 kg/tree) + BC (4.0 kg/tree) + JG (0.5 kg/tree)	de	2.49	3.31	2.90
T_10_	PM (3 kg/tree) + BC (5.0 kg/tree) + JG (0.75 kg/tree)	h	1.49	2.73	2.11
T_11_	PM (3 kg/tree) + BC (6.0 kg/tree) + JG (1.0 kg/tree)	e	1.93	3.69	2.81
T_12_	FM (15 kg/tree) (100% replacement of N through FYM)	j	1.61	1.64	1.63
T_13_	FM (10 kg/tree) + CU (0.75 liter/tree) + NC (1.5 kg/tree)	f	2.28	3.01	2.64
T_14_	FM (10 kg/tree) + CU (1.25 liter/tree) + NC (1.75 kg/tree)	f	1.93	3.21	2.57
T_15_	FM (10 kg/tree) + CU (1.75 liter/tree) + NC (2.0 kg/tree)	i	1.64	2.03	1.84
T_16_	Recommended POP (Control)	g	1.68	2.85	2.26
S.E. m(±)			0.01	0.09	0.01
*CD (0.05)*			0.03	0.26	0.13

*CD (0.05)*, Critical Difference at 5%; S.E.m (±), Standard error of mean.

**Table:** Analysis of variance for non-reducing sugars (%).

Source of variation	df	Sum of squares	Mean square	F value	P value
Treatment	15	39.713	2.6475	212.23	<0.001 ***
Replication	2	0.025	0.0125	1.00	0.3733 ns
Environment (Year)	1	5.185	5.1848	415.63	<0.001 ***
Treatment × Environment	15	25.675	1.7117	137.21	<0.001 ***
Error	62	0.773	0.0125	—	—
Total	95	71.371	—	—	—

*** → Highly significant at P ≤ 0.001

ns → Non-significant.

### 3.5 Titratable acidity (%)

The data on titratable acidity (%) influenced by the usage of different organic nutrient sources are presented in **Table 8** and was assessed across two growing seasons, 2022–2023 and 2023-2024, with pooled data provided a comprehensive overview of the treatment effects. In the year 2022-2023, the lowest acidity was found in treatment T_11_ (0.21%), which *at par with T_6_ (0.22*%*)* as compared to all treatments tried. Highest titratable acidity was recorded in treatment T_3_ (0.44%) as compared to control T_16_ (0.41%), which was at par with treatment T_3_. The trends in 2023–2024 largely differed those of the previous year. Treatment T_6_ (0.26%) achieved the significant lowest titratable acidity, which at par with treatment T_5_ (0.27%), as compared to all applied treatments.%. After combined the data from both years, treatment T_6_ (0.24%) showed the lowest titratable acidity, which at par with treatment T_5_ (0.26%) and T_4_ (0.29%). The highest value in titratable acidity were noted in treatment T_16_ (0.43%) (control).

**Table 8:** Effect of organic sources of nutrients on titratable acidity (%) of guava cv. L-49.

Treatments		Grouping (α = 0.05)	Titratable acidity (%)		
			2022–2023	2023–2024	Pooled
T_1_	VC (15 kg/tree) (100% replacement of N through VC)	bc	0.39	0.36	0.38
T_2_	VC (10 kg/tree) + CU (0.5 liter/tree) + NC (1.0 kg/tree)	de	0.33	0.31	0.32
T_3_	VC (10 kg/tree) + CU (1.0 liter/tree) + NC (1.5 kg/tree)	ab	0.44	0.35	0.39
T_4_	VC (10 kg/tree) + CU (1.5 kg/tree) + NC (2.0 kg/tree)	ef	0.29	0.29	0.29
T_5_	VC (5 kg/tree) + BC (5.0 kg/tree) + JG (1.0 kg/tree)	fg	0.25	0.27	0.26
T_6_	VC (5 kg/tree) + BC (7.5 kg/tree) + JG (1.25 kg/tree)	g	0.22	0.26	0.24
T_7_	VC (5 kg/tree) + BC (10 kg/tree) + JG (1.50 kg/tree)	de	0.33	0.32	0.33
T_8_	PM (5 kg/tree) (100% replacement of N through PM)	bc	0.42	0.33	0.38
T_9_	PM (3 kg/tree) + BC (4.0 kg/tree) + JG (0.5 kg/tree)	cd	0.38	0.30	0.34
T_10_	PM (3 kg/tree) + BC (5.0 kg/tree) + JG (0.75 kg/tree)	de	0.33	0.28	0.31
T_11_	PM (3 kg/tree) + BC (6.0 kg/tree) + JG (1.0 kg/tree)	ef	0.21	0.37	0.29
T_12_	FM (15 kg/tree) (100% replacement of N through FYM)	bcd	0.33	0.38	0.35
T_13_	FM (10 kg/tree) + CU (0.75 liter/tree) + NC (1.5 kg/tree)	bc	0.42	0.34	0.38
T_14_	FM (10 kg/tree) + CU (1.25 liter/tree) + NC (1.75 kg/tree)	de	0.27	0.39	0.33
T_15_	FM (10 kg/tree) + CU (1.75 liter/tree) + NC (2.0 kg/tree)	ab	0.38	0.40	0.39
T_16_	Recommended POP (Control)	a	0.41	0.45	0.43
S.E.m (±)			0.01	0.03	0.00
*CD (0.05)*			0.03	0.10	0.05

*CD (0.05)*, Critical Difference at 5%; S.E.m (±), Standard error of mean.

**Table:** Analysis of variance for titrable acidity (%).

Source of variation	df	Sum of squares	Mean square	F value	P value
Treatment	15	0.260229	0.0173486	8.02	<0.001 ***
Replication	2	0.008152	0.0040760	1.88	0.1607 ns
Environment (Year)	1	0.000150	0.0001500	0.07	0.7932 ns
Treatment × Environment	15	0.116883	0.0077922	3.60	<0.001 ***
Error	62	0.134181	0.0021642	—	—
Total	95	0.519595	—	—	—

*** → Highly significant at P ≤ 0.001

ns → Non-significant

### 3.6 Vitamin C (mg 100 g^−1^ pulp)

The data on vitamin C content during both years presented in **Table 9**. Significant differences were noted among the treatments in terms of vitamin C levels. The synergistic effect of organic nutrient sources on vitamin C (mg 100 g^−1^ pulp pulp) of guava was noted over two years, 2022–2023 and 2023-2024, with pooled data showing the combined effect of the treatments. In the year 2022-2023, the significant highest vitamin C (mg 100 g^−1^ pulp pulp) was recorded in T_6_ (173.57 mg 100 g^−1^ pulp *), followed by T_7_ (172.88* mg 100 g^−1^ pulp *)* in comparison with control T_16_ (146.86 mg 100 g^−1^ pulp ) had a lowest noted value of vitamin. In the year 2023-2024, the trends remained consistent, with T_6_ (176.97 mg 100 g^−1^ pulp ) again showing the significant maximum value, followed by treatment T_7_ (175.11 mg 100 g^−1^ pulp ) over control T_16_ (149.16 mg/100g ) which had the minimum value in vitamin C. When the results from both years were consolidated, it was showed that the significant highest vitamin C was found in treatment T_6_ (175.27 mg 100 g^−1^ pulp), which at par with T_7_ (173.99 mg 100 g^−1^ pulp ) as compared to control T_16_ (148.01 mg 100 g^−1^ pulp ), which showed the lowest vitamin C content.

**Table 9:** Effect of organic sources of nutrients on vitamin C (mg 100 g^−1^ pulp ) of guava cv. L-49.

Treatments		Grouping (α = 0.05)	Vitamin C (mg 100 g^−1^ pulp )		
			2022–2023	2023–2024	Pooled
T_1_	VC (15 kg/tree) (100% replacement of N through VC)	e	161.54	163.94	162.74
T_2_	VC (10 kg/tree) + CU (0.5 liter/tree) + NC (1.0 kg/tree)	gh	155.80	162.25	159.02
T_3_	VC (10 kg/tree) + CU (1.0 liter/tree) + NC (1.5 kg/tree)	fg	162.21	163.08	162.65
T_4_	VC (10 kg/tree) + CU (1.5 kg/tree) + NC (2.0 kg/tree)	d	163.49	168.38	165.94
T_5_	VC (5 kg/tree) + BC (5.0 kg/tree) + JG (1.0 kg/tree)	bc	171.42	172.09	171.75
T_6_	VC (5 kg/tree) + BC (7.5 kg/tree) + JG (1.25 kg/tree)	a	173.57	176.97	175.27
T_7_	VC (5 kg/tree) + BC (10 kg/tree) + JG (1.50 kg/tree)	ab	172.88	175.11	173.99
T_8_	PM (5 kg/tree) (100% replacement of N through PM)	h	153.82	155.77	154.80
T_9_	PM (3 kg/tree) + BC (4.0 kg/tree) + JG (0.5 kg/tree)	de	159.84	168.31	164.07
T_10_	PM (3 kg/tree) + BC (5.0 kg/tree) + JG (0.75 kg/tree)	de	161.23	169.04	165.14
T_11_	PM (3 kg/tree) + BC (6.0 kg/tree) + JG (1.0 kg/tree)	c	168.61	172.54	170.58
T_12_	FM (15 kg/tree) (100% replacement of N through FYM)	gh	153.42	157.31	155.37
T_13_	FM (10 kg/tree) + CU (0.75 liter/tree) + NC (1.5 kg/tree)	fgh	155.00	159.09	157.05
T_14_	FM (10 kg/tree) + CU (1.25 liter/tree) + NC (1.75 kg/tree)	gh	151.64	160.15	155.90
T_15_	FM (10 kg/tree) + CU (1.75 liter/tree) + NC (2.0 kg/tree)	fg	156.52	158.63	157.58
T_16_	Recommended POP (Control)	i	146.86	149.16	148.01
S.E.m (±)			1.39	1.23	5.33
*CD (0.05)*			4.04	3.57	2.66

*CD (0.05)*, Critical Difference at 5%; S.E.m (±), Standard error of mean.

**Table:** Analysis of variance for vitamin C (mg 100 g^−1^ pulp).

Source of Variation	df	Sum of squares	Mean square	F value	P value
Treatment	15	5383.8	358.92	67.26	<0.001 ***
Replication	2	11.7	5.83	1.09	0.3417 ns
Environment (Year)	1	383.8	383.76	71.91	<0.001 ***
Treatment × Environment	15	148.9	9.93	1.86	0.0457 *
Error	62	330.9	5.34	—	—
Total	95	6259.1	—	—	—

*→ Significant at P ≤ 0.05

*** → Highly significant at P ≤ 0.001

ns → Non-significant

### 3.7 Pectin content (%)

The data pertaining to pectin content in fruits of guava showed a significant difference during both the years as well as in pooled estimates between treatments (**Table 10**). The influence of organic nutrient sources on guava pectin content (%) was recorded over two growing seasons, 2022–2023 and 2023-2024, with pooled data providing an overall assessment of treatment effects. In 2022–2023 year, the significant highest pectin content was observed in T_14_ (0.97%), which at par with treatment T_8_ (0.94%) as compared to control T_16_ (0.53%). In the year 2023-2024, the trend remained largely differed, with T_6_ (1.03%) producing the significant highest pectin content, at par with T_11_ (1.01%) and T_12_ (1.00%) as compared to control T_16_ (0.58%). When data from both years were pooled, T_7_ (0.96%) and T_14_ (0.96%) emerged as the top-performing treatment, respectively which at par with treatment T_1_ (0.94%) and followed by treatment T_4_ (0.93%) and T_9_ (0.93%) as compared to control T_16_ (0.56%). The variation in pectin content across years suggests that the response of this parameter to organic nutrient sources may be influenced by seasonal or environmental factors. Therefore, while treatment T_7_ recorded the highest pooled pectin content, the year-to-year variability indicates that differences among treatments should be interpreted with caution.

**Table 10:** Effect of organic sources of nutrients on pectin content (%) of guava cv. L-49.

Treatments		Grouping (α = 0.05)	Pectin content (%)		
			2022–2023	2023–2024	Pooled
T_1_	VC (15 kg/tree) (100% replacement of N through VC)	B	0.92	0.97	0.94
T_2_	VC (10 kg/tree) + CU (0.5 liter/tree) + NC (1.0 kg/tree)	E	0.83	0.89	0.86
T_3_	VC (10 kg/tree) + CU (1.0 liter/tree) + NC (1.5 kg/tree)	F	0.77	0.78	0.78
T_4_	VC (10 kg/tree) + CU (1.5 kg/tree) + NC (2.0 kg/tree)	B	0.92	0.93	0.93
T_5_	VC (5 kg/tree) + BC (5.0 kg/tree) + JG (1.0 kg/tree)	D	0.87	0.89	0.88
T_6_	VC (5 kg/tree) + BC (7.5 kg/tree) + JG (1.25 kg/tree)	D	0.76	0.99	0.88
T_7_	VC (5 kg/tree) + BC (10 kg/tree) + JG (1.50 kg/tree)	A	0.88	1.03	0.96
T_8_	PM (5 kg/tree) (100% replacement of N through PM)	C	0.94	0.87	0.91
T_9_	PM (3 kg/tree) + BC (4.0 kg/tree) + JG (0.5 kg/tree)	B	0.87	0.99	0.93
T_10_	PM (3 kg/tree) + BC (5.0 kg/tree) + JG (0.75 kg/tree)	E	0.93	0.77	0.85
T_11_	PM (3 kg/tree) + BC (6.0 kg/tree) + JG (1.0 kg/tree)	C	0.79	1.01	0.90
T_12_	FM (15 kg/tree) (100% replacement of N through FYM)	B	0.85	1.00	0.93
T_13_	FM (10 kg/tree) + CU (0.75 liter/tree) + NC (1.5 kg/tree)	H	0.75	0.72	0.74
T_14_	FM (10 kg/tree) + CU (1.25 liter/tree) + NC (1.75 kg/tree)	A	0.97	0.95	0.96
T_15_	FM (10 kg/tree) + CU (1.75 liter/tree) + NC (2.0 kg/tree)	G	0.75	0.76	0.76
T_16_	Recommended POP (Control)	I	0.53	0.58	0.56
S.E.m (±)			0.01	0.01	0.00
*CD (0.05)*			0.03	0.03	0.02

*CD (0.05)*, Critical Difference at 5%; S.E.m (±), Standard error of mean.

**Table:** Analysis of variance for pectin content (%).

Source of variation	df	Sum of squares	Mean square	F value	P value
Treatment	15	1.02503	0.068335	210.35	<0.001 ***
Replication	2	0.00032	0.000162	0.50	0.6088 ns
Environment (Year)	1	0.06100	0.061004	187.78	<0.001 ***
Treatment × Environment	15	0.24896	0.016597	51.09	<0.001 ***
Error	62	0.02014	0.000325	—	—
Total	95	1.35545	—	—	—

*** → Highly significant at P ≤ 0.001

ns → Non-significant

## 4 Discussion

In general discussion, vermicompost provides essential macro- and micronutrients and may enhance beneficial microbial activity, which could improve nutrient availability and sugar accumulation in fruits. Biochar, as a stable carbon-rich material, improved soil structure, water retention, and created favorable microhabitats for soil microbes, ensuring sustained nutrient availability. Jaggery, being rich in soluble sugars and micronutrients, likely served as a quick energy source for soil microbes, promoting decomposition of organic matter and indirectly enhancing sugar accumulation in the fruits.

In the present study, total soluble solids (%) are significantly affected by the application of organic nutrient sources comprising of 5 kg of vermicompost per tree, 7.5 kg of biochar per tree and 1.25 kg of jaggery per tree during both the years. These results possibly caused by the application of a biochar-vermicompost complex with jaggery has shown to significantly increase the Total Soluble Solids (TSS) in guava fruit. This effect resulted in several synergistic processes enabled by the complex. Biochar, a stable form of carbon, improves structure of soil and enhances nutrient retention. When combined with vermicompost, which is rich in organic matter and beneficial microorganisms, this complex creates an optimal environment for plant growth and nutrient availability. Vermicompost contributes essential nutrients and may also contain plant growth-promoting substances, while biochar can improve nutrient retention and provide favorable habitats for soil microorganisms (Lehmann, 2007). During photosynthesis, sucrose is produced in the leaves and transported to the developing fruits via the phloem. Once in the fruit, sucrose is metabolized into simpler sugars, such as glucose and fructose, through the action of enzymes like invertase and sucrose synthase. As the fruit ripens, organic acids, which are abundant in immature fruits, decrease due to metabolic conversion into sugars or dilution, enhancing the sweetness (Falchi et al., 2020). Fruit ripening generally involves the conversion of sucrose into simpler sugars through enzymatic processes, which are regulated by plant hormones such as ethylene. Although these processes were not directly measured in the present study, they may partly involve in increase in TSS (Gautam et al., 2022). This was improved by nutrient availability leads to enhanced metabolic processes within the guava crop, particularly the conversion of starches and insoluble carbohydrates into soluble sugars, which directly increases TSS in the fruit (Singh et al., 2020). Studies have demonstrated that such organic soil amendments can lead to significant improvements in fruit quality, as evidenced by increased TSS in guava. The application of biochar significantly enhanced the soluble solids content, resulting in greater sweetness compared to fruits grown without biochar (Villocino and Quevedo, 2015). The biochar-vermicompost complex with combination of jaggery thus represents an effective approach for enhancing fruit quality through both nutrient management and microbial stimulation. The earlier findings of Goswami et al. (2015); Kumrawat et al. (2018) and Tyagi et al. (2018) in guava are also in consonance with the present results.

Notably, it is clear that different types of organic manure had a significant impact on total sugars, reducing sugars and non-reducing sugars. The significant increase in total sugars were recorded in treatment comprising of vermicompost at 5 kg/tree combined with biochar at 7.5 kg/tree and jaggery at 1.25 kg/tree and significant maximum reducing sugar were found in treatment comprising of vermicompost at 5 kg/tree with Biochar further increased to 10 kg/tree alongside Jaggery at 1.50 kg/tree and non-reducing sugar were observed in treatment comprising of vermicompost at 5 kg/tree with biochar increased to 5 kg/tree and jaggery at 1 kg/tree sugar during both the years. These incremental increases in biochar and jaggery were designed to assess their synergistic influence on soil properties, microbial activity, and nutrient availability, thereby optimizing fruit growth and yield parameters in guava. This enhancement may be attributed to the synergistic effect of vermicompost, biochar, and jaggery, which collectively contribute to a significant increase in total, reducing, and non-reducing sugars in guava fruits. The observed improvement in sugar content under the combined application of these amendments could result from their ability to enhance soil structure, boost nutrient availability, improve water retention, and promote better soil aeration. Vermicompost, in particular, is rich in essential amino acids like glutamic acid and glycine, which play a crucial role in metabolic activities associated with sugar accumulation. These amino acids are readily absorbed by plant roots, thereby facilitating the synthesis and accumulation of soluble sugars in the fruits (Wang et al., 2010; Scaglia et al., 2016; Zhang et al., 2023). Furthermore, the accumulation of secondary metabolites, such as β-carotenoids, total carbohydrate, polyphenols and antioxidants, in guava fruits increased due to improvements in the photosynthesis system of the trees (El-Beltagi et al., 2023; Abdeldaym et al., 2024). Similarly, the positive effect of vermicompost might be described by the fact that vermicompost increases essential amino acids in plants, which may play a role in the regulation of sugar metabolism and endorse the improvement of total sugar content in fresh fruits [34]. Biochar, with its stable carbon structure, improves soil structure and nutrient retention, fostering a conducive environment for plant growth. It may enhances nutrient availability and microbial activity in the soil, which are crucial for the efficient conversion of complex substances like starch into simple sugars, thereby increasing the sugar in guava. Jaggery may stimulate microbial activity in the soil, which could enhance nutrient availability and potentially influence plant growth processes. This combination leads to increased nutrient uptake, improved catalytic activities, and the degradation of starch into simple sugars, resulting in higher total, reducing and non-reducing sugar content in guava. These observations align with the findings of Bhobia et al. (2005) and Shukla et al. (2009)in guava, and Baraily and Deb (2018) in pineapple. Comparable results have been reported by various researchers, indicating that the combined application of vermicompost, compost, biochar, and jaggery enhances both the quantity and quality of guava fruits, particularly by increasing levels of total, reducing, and non-reducing sugars (Mahmud et al., 2021; Liu et al., 2022; Taşcı and Kuzucu, 2023; Zhang et al., 2023).

The present study indicated that the titratable acidity content in guava exhibited significant variation in response to the application of different organic manures across both the years. Titratable acidity is another important parameter, reflecting the fruit's overall tartness and acidity profile. The data shows that titratable acidity varied between treatments and across years. The control exhibited the significant highest titratable acidity. It has been observed that an increase in total soluble solids (TSS) correlates with a decrease in acidity. Lodaya and Masu (2019) and Singh et al. (2008)found that the highest TSS also exhibited and the lowest acidity was registered in guava and strawberry. This reduction in acidity may be attributed to the utilization of organic acids in metabolic processes such as respiration and biodegradation (Ulrich, 1970). The reduction in titratable acidity observed during the later stages of fruit maturation and ripening is likely a result of the transformation of organic acids into sugars. The beneficial effect of vermicompost on total soluble solids (TSS) can be attributed to its high content of essential amino acids, particularly glutamic acid and glycine. These amino acids are absorbed by plant roots and participate in metabolic pathways related to soluble sugar synthesis, thereby contributing to increased sugar accumulation in the fruits (Wang et al., 2010; Scaglai et al., 2016).

Another parameter, also it was evident from the present investigation that vitamin C content varied significantly in response to the application of different organic sources of nutrients comprising of 5 kg of vermicompost per tree, 7.5 kg of biochar per tree and 1.25 kg of jaggery per tree during the both years. The increase in vitamin C content may be associated with improved nutrient availability and metabolic activity in plants under organic nutrient management. This finding aligns with the studies by Umar et al. (2009); ); Singh et al. (2018); Tyagi et al. (2018 in strawberry, which revealed significant variations in ascorbic acid (vitamin C) concentrations among sweet potato roots grown under different organic amendments, including biochar, also effectively promoted vitamin C concentration. Furthermore, the synergistic benefits of biochar and vermicompost in improving the quality of continuously cropped pepper were noted, possibly due to the complementary effects of biochar and vermicompost. Vermicompost supplies nutrients, while biochar enhances cation exchange capacity and long-term carbon fixation (Álvarez et al., 2018; Hafez et al., 2020; Kumari et al.., 2020; Ebrahimi et al., 2021; Wang et al., 2021) . The increase in ascorbic acid content due to various organic treatments may result from improved plant nutrition, enhancing vitamins and minerals in fruits. These results closely match the findings of Shukla et al. (2014) and Goswami et al. (2015b).

An inquisition of the data in this study reveals that the pectin content of guava was significantly influenced by the increased level of soil nutrients through different types of organic amendments over both the years. Maximum pectin content was recorded with the application of organic manure comprising 5 kg of vermicompost (prepared from well-decomposed cow dung and agro-residues using *Eisenia fetida* earthworms) per tree, 10 kg of biochar per tree, and 1.50 kg of jaggery per tree. The enhancement of pectin content in guava through the application of organic amendments can be explained by their synergistic effects on soil health, nutrient availability, and plant physiological processes. Biochar, a highly porous carbon-rich material produced via pyrolysis, improves soil structure by increasing aeration, water retention and the nutrient-holding capacity of the soil. The porous structure of biochar aids in the retention of essential nutrients such as nitrogen, phosphorus, and potassium by reducing nutrient leaching and ensuring their sustained availability to plants. Furthermore, biochar supports enhanced microbial activity in the soil by offering a favorable habitat for beneficial microorganisms, which play a critical role in nutrient cycling and the decomposition of organic matter. Enhanced soil microbial activity under organic amendments may influence plant metabolic processes, which could contribute to pectin accumulation in fruits. In parallel, vermicompost formed through the breakdown of organic material by earthworms is a rich source of both macro- and micronutrients, such as nitrogen, phosphorus, potassium, calcium, and magnesium, along with a diverse population of beneficial microorganisms. Vermicompost contributes to improving soil fertility, organic matter content, and the microbial diversity in the rhizosphere. The microbial populations in vermicompost, including phosphate-solubilizing bacteria (PSB) and nitrogen-fixing bacteria like Azotobacter, enhance nutrient availability, particularly phosphorus and nitrogen, both of which are vital for plant growth and biochemical processes, including the biosynthesis of pectin (Binepal et al., 2013). Furthermore, earthworms contribute to improved soil structure and aeration, facilitating root penetration and nutrient uptake, which can indirectly support the metabolic processes involved in pectin production. These essential nutrients play a significant role in improving quality as reported by Srinil et al. (2020). BiNepal et al. (2013) in guava also noted similar results.

## 5 Conclusion

Based on experimental results, the treatment T**_6_**recorded comparatively higher values for several biochemical parameters; however, some treatments, particularly T**_7_**, were statistically at par for certain attributes. Therefore, these treatments may be considered promising organic nutrient combinations for improving guava fruit quality under subtropical conditions.

## Data Availability

The original contributions presented in the study are included in the article/supplementary material. Further inquiries can be directed to the corresponding authors.

## Appendix 1

**Table:** Monthly meteorological parameters recorded at the experimental site (SKUAST-Jammu) during the study period 2022–2024.

Standard meteorological months	Temperature (°C)		Relative humidity (morning)	Relative humidity (evening)	Rainfall (mm)
	Maximum	Minimum			
October, 2022-23	28.92	14.80	79.24	45.76	34.00
November, 2022-23	26.20	8.26	90.72	36.75	–
December, 2022-23	20.96	4.96	91.69	47.43	0.20
January, 2023	16.16	7.41	92.29	72.97	40.24
February, 2023	22.85	7.57	90.14	51.54	7.24
March, 2023	35.20	15.62	70.37	32.05	–
April, 2023	38.88	20.80	48.52	22.44	1.72
May, 2023	38.22	23.06	53.66	30.58	19.28
October, 2023-24	30.23	14.60	84.50	43.77	0.80
November, 2023-24	25.05	9.30	91.13	48.45	6.20
December,2023-24	20.93	5.55	92.95	51.90	2.40
January, 2024	16.62	5.14	94.60	70.00	10.36
February, 2024	25.98	9.42	88.40	47.20	0.12
March, 2024	28.42	13.58	91.43	48.11	24.92
April, 2024	33.78	17.92	65.57	33.00	18.68
May, 2024	36.02	22.76	71.77	42.14	20.00
